# Research diagnostic criteria for Alzheimer’s disease: findings from the LipiDiDiet randomized controlled trial

**DOI:** 10.1186/s13195-021-00799-3

**Published:** 2021-03-25

**Authors:** Anna Rosenberg, Alina Solomon, Hilkka Soininen, Pieter Jelle Visser, Kaj Blennow, Tobias Hartmann, Miia Kivipelto, on behalf of the LipiDiDiet clinical study group, Hilkka Soininen, Ilona Hallikainen, Merja Hallikainen, Seppo Helisalmi, Tarja Lappalainen, Yawu Liu, Teemu Paajanen, Miia Kivipelto, Alina Solomon, Lars-Olof Wahlund, Yvonne Freund-Levi, Göran Hagman, Kaj Blennow, Tobias Hartmann, Klaus Fassbender, Matthias Riemenschneider, Marcus O. W. Grimm, Aline Klees-Rollmann, Maxine Luley, Epameinondas Lyros, Robert Schomburg, Daniela Ramelli, Jennifer Kennel, Lutz Frölich, Lucrezia Hausner, Christoph Laske, Thomas Leyhe, Christian Mychajliw, Niklas Koehler, Stephan Schiekofer, Hans Klünemann, Johannes Schröder, Dieter Lütjohann, Pieter Jelle Visser, Philip Scheltens, Ineke van Rossum, Nienke Scheltens, Daniela Bertens, Mara ten Kate, Frederik Barkhof, Silvia Ingala, Johanna M. L. Henselmans, Gerwin Roks, Anneke M. J. van Hees, Floor M. van Oudenhoven, Suzanne B. Hendrix, Noel Ellison

**Affiliations:** 1 Department of Neurology, Institute of Clinical Medicine, University of Eastern Finland, Kuopio, Finland; 2 Division of Clinical Geriatrics, Centre for Alzheimer Research, Department of Neurobiology, Care Sciences and Society, Karolinska Institutet, Stockholm, Sweden; 3 Theme Aging, Karolinska University Hospital, Stockholm, Sweden; 4 Neurocenter, Department of Neurology, Kuopio University Hospital, Kuopio, Finland; 5 Department of Psychiatry and Neuropsychology, Alzheimer Centre Limburg, University of Maastricht, Maastricht, Netherlands; 6 Department of Neurology, Alzheimer Centre, Amsterdam Neuroscience, VU University Medical Centre, Amsterdam, Netherlands; 7 Department of Psychiatry and Neurochemistry, Institute of Neuroscience and Physiology, The Sahlgrenska Academy at University of Gothenburg, Mölndal, Sweden; 8 Clinical Neurochemistry Laboratory, Sahlgrenska University Hospital, Mölndal, Sweden; 9 Deutsches Institut für Demenz Prävention (DIDP), Medical Faculty, and Department of Experimental Neurology, Saarland University, Homburg, Germany; 10 Institute of Public Health and Clinical Nutrition, University of Eastern Finland, Kuopio, Finland; 11 Ageing Epidemiology Research Unit, School of Public Health, Imperial College London, London, UK

**Keywords:** Alzheimer’s disease, Prodromal Alzheimer’s disease, Randomized controlled trial, Prevention, Disease progression, Research criteria, Early diagnosis, Biomarkers, Cerebrospinal fluid

## Abstract

**Background:**

To explore the utility of the International Working Group (IWG)-1 criteria in recruitment for Alzheimer’s disease (AD) clinical trials, we applied the more recently proposed research diagnostic criteria to individuals enrolled in a randomized controlled prevention trial (RCT) and assessed their disease progression.

**Methods:**

The multinational LipiDiDiet RCT targeted 311 individuals with IWG-1 defined prodromal AD. Based on centrally analyzed baseline biomarkers, participants were classified according to the IWG-2 and National Institute on Aging–Alzheimer’s Association (NIA-AA) 2011 and 2018 criteria. Linear mixed models were used to investigate the 2-year change in cognitive and functional performance (Neuropsychological Test Battery NTB *Z* scores, Clinical Dementia Rating-Sum of Boxes CDR-SB) (criteria × time interactions; baseline score, randomization group, sex, Mini-Mental State Examination (MMSE), and age also included in the models). Cox models adjusted for randomization group, MMSE, sex, age, and study site were used to investigate the risk of progression to dementia over 2 years.

**Results:**

In total, 88%, 86%, and 69% of participants had abnormal cerebrospinal fluid (CSF) β-amyloid, total tau, and phosphorylated tau, respectively; 64% had an A+T+N+ profile (CSF available for *N* = 107). Cognitive-functional decline appeared to be more pronounced in the IWG-2 prodromal AD, NIA-AA 2011 high and intermediate AD likelihood, and NIA-AA 2018 AD groups, but few significant differences were observed between the groups within each set of criteria. Hazard ratio (95% CI) for dementia was 4.6 (1.6–13.7) for IWG-2 prodromal AD (reference group no prodromal AD), 7.4 (1.0–54.7) for NIA-AA 2011 high AD likelihood (reference group suspected non-AD pathology SNAP), and 9.4 (1.2–72.7) for NIA-AA 2018 AD (reference group non-Alzheimer’s pathologic change). Compared with the NIA-AA 2011 high AD likelihood group (abnormal β-amyloid and neuronal injury markers), disease progression was similar in the intermediate AD likelihood group (medial temporal lobe atrophy; no CSF available).

**Conclusions:**

Despite being less restrictive than the other criteria, the IWG-1 criteria reliably identified individuals with AD pathology. More pragmatic and easily applicable selection criteria might be preferred due to feasibility in certain situations, e.g., in multidomain prevention trials that do not specifically target β-amyloid/tau pathologies.

**Trial registration:**

Netherlands Trial Register, NL1620. Registered on 9 March 2009

## Background

To decelerate cognitive decline and delay dementia onset in Alzheimer’s disease (AD), preventive interventions may need to target high-risk individuals early in the disease course. Several research diagnostic criteria relying on biomarker evidence for β-amyloid (Aβ) and neuronal injury have been proposed to identify such individuals for randomized controlled trials (RCTs) [1–7]. Compared to biomarker-positive individuals, more heterogeneous groups of individuals and those without confirmed AD pathology may have a lower likelihood of cognitive decline [8]. A lower than expected decline in the placebo group combined with a lower than expected effect in the intervention group could mask beneficial treatment effects in RCTs. Indeed, past RCT failures might partly be attributable to suboptimal participant selection [9, 10].

The International Working Group (IWG)-1 research criteria for AD [1] attribute equal weight to all biomarkers, whereas the newer criteria emphasize Aβ [3–7]. Most ongoing prodromal AD RCTs also require Aβ positivity [11]. Biomarker-focused selection criteria and their impact on trial design have been investigated in simulation studies [11–16], but little is known about the operationalization of these criteria in real-life RCTs. Given that the more recently proposed criteria require comprehensive biomarker assessments, it is also relevant to study the potential utility of IWG-1 and other more easily applicable criteria in the recruitment for prevention RCTs. The multinational LipiDiDiet RCT, which investigated the effects of medical food on cognition in prodromal AD, was one of the first RCTs using the IWG-1 criteria in participant selection [17]. To understand whether these criteria can be successfully applied to identify individuals with AD biomarker profiles, we investigated LipiDiDiet participants’ baseline biomarker profiles and classified them according to all other currently available research criteria: the IWG-2, National Institute on Aging–Alzheimer’s Association (NIA-AA) 2011, and NIA-AA 2018 criteria. Furthermore, to understand the impact of biomarker profile on disease progression, we assessed the 2-year change in cognitive and functional performance as well as progression to dementia in the groups within each set of criteria.

## Methods

### Trial design and participants

LipiDiDiet is a double-blind proof-of-concept RCT conducted at 11 sites in Finland, Sweden, Germany, and the Netherlands [17]. The 2-year core trial, completed in 2015, was followed by up to four 1-year double-blind extension studies. LipiDiDiet included 311 individuals aged 55–85 years, recruited mainly from memory clinics. For eligibility, participants were required to meet the IWG-1 criteria for prodromal AD [1], i.e., have an underlying AD pathology and mild episodic memory impairment, defined in LipiDiDiet as a performance below one standard deviation in at least two cognitive tests (at least one memory test). The following cognitive screening tests were used: Free and Cued Selective reminding test, free and delayed recall, Wechsler Memory Scale-revised (WMS-r) story, delayed recall, WMS-r delayed recall of figures, Trail Making Tests A and B, symbol digit substitution test, and category fluency test. Sufficient evidence for AD pathology was defined as abnormality in at least one of the following biomarkers: cerebrospinal fluid (CSF) Aβ (Aβ42 and/or Aβ42/40 ratio), CSF total tau (t-tau), CSF phosphorylated tau (p-tau), fluoro-deoxy-glucose-positron emission tomography (FDG-PET), or medial temporal lobe atrophy (MTA) on magnetic resonance imaging (MRI). Due to feasibility, MTA was often the primary biomarker used to assess eligibility in LipiDiDiet.

Exclusion criteria were dementia diagnosis or substantial cognitive impairment (Mini-Mental State Examination (MMSE) < 24 or < 20 if ≤ 6 years of education), cholinesterase inhibitor or memantine use, neuroimaging abnormalities (stroke, intracranial bleeding, mass lesion, normal pressure hydrocephalus), and conditions potentially interfering with participation (e.g., alcohol/drug abuse). Individuals taking omega-3 products or vitamin B6, B12, C, E, or folic acid (> 200% of the recommended daily intake) were also excluded due to the nature of the intervention.

LipiDiDiet is registered at Netherlands Trial Register (identifier NL1620). Ethical approval was granted by local ethics committees, and written informed consent was obtained from all participants and study partners prior to enrollment.

### Trial protocol

Participants were randomized in a 1:1 ratio to the intervention and control groups. The intervention group consumed once a day a 125-ml medical food drink (Souvenaid®) containing the multinutrient Fortasyn Connect™ (Nutricia; Zoetermeer, the Netherlands). Fortasyn Connect™ consists of a combination of nutrients which AD patients might be deficient in [18], including omega-3 fatty acids, vitamins, folic acid, phospholipids, and antioxidants. In short-term RCTs in mild AD, this multinutrient showed beneficial effects on cognitive performance and brain functional connectivity [19, 20]. The control group consumed once a day an iso-caloric placebo product similar in appearance, flavor, and composition, but without the multinutrient. The participants, clinical team, and outcome evaluators were blinded to group assignment. Main study parameters and outcomes were assessed at baseline, 6, 12, and 24 months. For motivation and compliance, additional visits with the study nurse or physician were organized at 3, 9, and 18 months, and phone calls were conducted monthly for the first 6 months and every 2 months after that. Protocol details are described elsewhere [17].

### Cognitive outcomes and progression to dementia

The primary trial outcome was change in cognitive performance measured with a Neuropsychological Test Battery (NTB) composite *Z* score including five tests: Consortium to Establish a Registry for Alzheimer’s Disease (CERAD) 10-word list learning, delayed recall, and recognition, category fluency test, and letter digit substitution test. Secondary cognitive outcomes included the domain-specific *Z* scores for memory (three tests; CERAD 10-word list learning, delayed recall, and recognition) and executive functioning (four tests; category fluency test, letter digit substitution test, concept shifting test condition C, and WMS-r digit span), as well as the NTB total *Z* score. The total score was based on 16 tests (the above-mentioned tests and additionally WMS-r logical verbal memory, immediate and delayed recall, WMS-r visual paired associates, immediate and delayed recall, 30-item Boston Naming Test, CERAD constructional praxis, copy and recall, and concept shifting test conditions A and B). Test scores were calculated as standardized *Z* scores with higher scores demonstrating better performance. Cognition was assessed by study psychologists at baseline, 6, 12, and 24 months.

Other secondary outcomes, including the Clinical Dementia Rating-Sum of Boxes (CDR-SB) score reflecting global cognitive-functional performance, were assessed at baseline, 12, and 24 months. Dementia and AD were diagnosed using the Diagnostic and Statistical Manual of Mental Disorders 4th edition (DSM-IV) [21] and the National Institute of Neurological and Communicative Disorders and Stroke–Alzheimer’s Disease and Related Disorders Association (NINCDS-ADRDA) criteria [22].

### Biomarker assessments

A harmonized and optimized protocol for CSF sampling was followed at all sites. Lumbar puncture was performed in the morning and 10 ml CSF was tapped by gravity drip. Samples were centrifuged (2000*g*, 10 min, + 4 °C), aliquoted in polypropylene tubes, and stored at − 70–80 °C for centralized analysis. Samples were first analyzed locally or sent for central laboratory analysis to assess eligibility at screening (pre-specified cut-offs for abnormality adjusted if necessary). For the evaluation of biomarker profiles and research diagnostic criteria, all samples, including those originally obtained for local analysis only, were analyzed or re-analyzed centrally in the Clinical Neurochemistry Laboratory at Sahlgrenska University Hospital, Mölndal, Sweden. Samples were handled by board-certified laboratory technicians and analyzed simultaneously using the same reagent batch, adhering to strict procedures for run acceptance and quality control procedures. Aβ40 and Aβ42 concentrations were measured using the MSD Abeta Triplex (Meso Scale Discovery, Rockville, Maryland) and the Aβ42/40 ratio was calculated as (Aβ42/Aβ40) × 10. T-tau and p-tau were measured using INNOTEST sandwich ELISAs (Fujirebio, Ghent, Belgium). The following cut-offs for CSF abnormality were applied in the LipiDiDiet central analysis and in the present study: Aβ42 < 450 pg/ml; Aβ42/40 ratio < 1; t-tau > 350 pg/ml; p-tau > 60 pg/ml.

At each study site, structural MRI was performed according to local scanning protocols. To assess MTA, coronal reconstructions of 3D T1-weighted scans were visually rated according to the Scheltens scale ranging from 0 (no atrophy) to 4 (severe atrophy). The right and left hemispheres were rated separately, and the sum was calculated. Scans were assessed locally to determine eligibility at screening. For the present study, results of the centralized analysis were used to assess biomarker profiles. Central analysis was conducted at the VU University Medical Center in Amsterdam, the Netherlands. MTA was defined as a score of ≥ 1.

### Classification according to the research diagnostic criteria

In the present study, all participants (both the intervention and control group participants) with centrally analyzed baseline biomarkers (CSF and/or MTA; *N* = 287) were classified according to the A/T/N biomarker scheme [23] and the IWG-2 [5], NIA-AA 2011 [3], and NIA-AA 2018 [7] research diagnostic criteria (Table 1). Biomarkers considered in the classification were MTA (imaging marker for neuronal injury) and CSF biomarkers reflecting Aβ deposition (Aβ42, Aβ42/40 ratio) and neuronal injury (p-tau, t-tau). Aβ or tau positron emission tomography (PET) scans were not performed in LipiDiDiet.

**Table 1:** Biomarker profiles and classification according to the research diagnostic criteria for AD

Criteria	Biomarker profile
**IWG-1—trial eligibility criteria**	
No prodromal AD	Normal CSF Aβ, t-tau, p-tau, MTA, FDG-PET
Prodromal AD	Abnormal CSF Aβ, t-tau, p-tau, MTA, and/or FDG-PET
**IWG-2**	
No prodromal AD	Normal CSF Aβ + normal/abnormal CSF t-tau, p-tau, MTA
Prodromal AD	Abnormal CSF Aβ + abnormal CSF t-tau and/or p-tau
**NIA-AA 2011**	
Low AD likelihood	Normal CSF Aβ + normal CSF t-tau, p-tau, and MTA
Isolate amyloid pathology	Abnormal CSF Aβ + normal CSF t-tau, p-tau, and MTA
Suspected non-AD pathology (SNAP)	Normal CSF Aβ + abnormal CSF t-tau, p-tau, and/or MTA
High AD likelihood	Abnormal CSF Aβ + abnormal CSF t-tau, p-tau, and/or MTA
Intermediate AD likelihood	CSF biomarkers not available, abnormal MTA
Inconclusive/uninformative	CSF biomarkers not available, normal MTA
**NIA-AA 2018**	
Normal AD biomarkers (A−T−N−)	Normal CSF Aβ + normal CSF t-tau, p-tau, and MTA
Alzheimer’s pathologic change (A+T−N−)	Abnormal CSF Aβ + normal CSF t-tau, p-tau, and MTA
Non-Alzheimer’s pathologic change (A−T+N±, A−T−N+)	Normal CSF Aβ + abnormal CSF p-tau, t-tau, and/or MTA
Alzheimer’s and concomitant suspected non-Alzheimer’s pathologic change (A+T−N+)	Abnormal CSF Aβ + normal CSF p-tau + abnormal CSF t-tau and/or MTA
AD (A+T+N±)	Abnormal CSF Aβ + abnormal CSF p-tau + normal/abnormal CSF t-tau and/or MTA

Abnormal CSF Aβ defined as Aβ42 < 450 pg/ml and/or Aβ42/40 × 10 < 1; abnormal CSF t-tau > 350 pg/ml; abnormal CSF p-tau > 60 pg/ml; abnormal MTA visual rating ≥ 1

*Abbreviations: Aβ*, β-amyloid; *AD*, Alzheimer’s disease; *CSF*, cerebrospinal fluid; *FDG-PET*, fluoro-deoxy-glucose-positron emission tomography; *IWG*, International Working Group; *MTA*, medial temporal lobe atrophy, visual rating; *NIA-AA*, National Institute on Aging-Alzheimer’s Association; *p-tau*, phosphorylated tau at threonine 181; *SNAP*, suspected non-AD pathology; *t-tau*, total tau

Participants were considered to have prodromal AD (IWG-2) if they had abnormal Aβ and at least one abnormal CSF neuronal injury marker. High AD likelihood (NIA-AA 2011) was defined as abnormal Aβ and at least one (any) abnormal neuronal injury marker. Participants with conflicting biomarkers were classified either in the isolate amyloid pathology group (abnormal Aβ but normal neuronal injury markers) or in the suspected non-AD pathology (SNAP) group (normal Aβ but at least one abnormal neuronal injury marker) [24]. Participants with only one biomarker available (in LipiDiDiet MTA) were classified as having either an intermediate AD likelihood or an inconclusive/uninformative status, depending on whether MTA was present. According to the NIA-AA 2018 criteria, participants with abnormal Aβ and p-tau were considered to have AD (A+T+N± profile). Participants with abnormal Aβ and neuronal injury markers but normal p-tau were classified in the Alzheimer’s and concomitant suspected non-Alzheimer’s pathologic change group (A+T−N+). In case of conflicting biomarkers, participants were classified as having an Alzheimer’s (A+T−N−) or non-Alzheimer’s pathologic change (A−T+N±, A−T−N+).

### Statistical analysis

Between-group differences in participant baseline characteristics were analyzed with *t*-tests and chi-square tests, as appropriate. *Z* scores for NTB composite, total, memory, and executive functioning were calculated as previously described [17]. We analyzed change from baseline in the NTB and CDR-SB scores using linear mixed models for repeated measures as previously reported, with baseline score, randomized treatment, time, treatment × time interaction, and baseline MMSE as fixed effects [17]. We additionally included the biomarker profile (research criteria) and criteria × time interaction, as well as sex and baseline age, as fixed effects in the present study. All trial participants regardless of randomized treatment were included in the analyses (intervention effects were accounted for by including randomized treatment and treatment × time interaction in the models). A random intercept with a variance components covariance structure was used within sites and a random intercept and slope for time with an unstructured covariance structure within subjects. Least-squares means for change from baseline were estimated from the linear mixed model. *p*-values are shown for the difference in least-squares means over 2 years between each group and the respective reference group.

Associations between the biomarker profile (research criteria) and 2-year risk of progression to dementia were analyzed with Cox proportional hazards models adjusted for randomized treatment (to account for intervention effects), baseline MMSE, sex, baseline age, and study site. Results are presented as hazard ratios (HR) and 95% CI. Results of all longitudinal analyses are reported for the modified intention-to-treat (mITT) population (all randomized participants with at least one post-baseline assessment, excluding visit data after progression to dementia and start of AD medication and/or open-label Souvenaid). SAS software version 9.4 was used in the analyses, and level of statistical significance was < 0.05. All analyses were post hoc.

## Results

### Baseline characteristics and classification according to the research diagnostic criteria

**Fig. 1:**
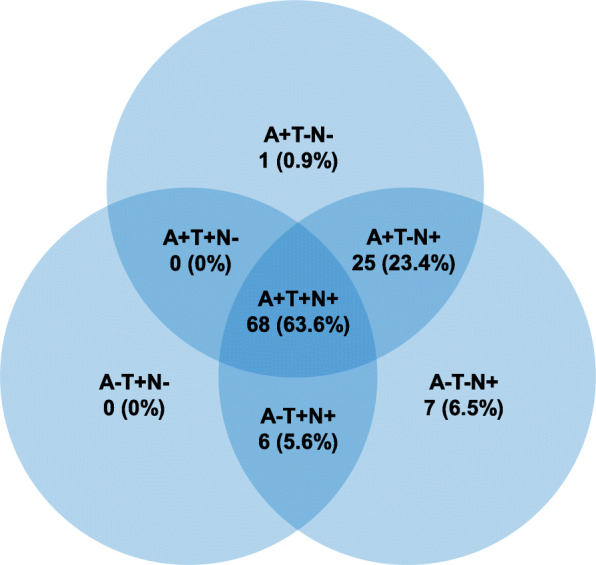
Baseline biomarker profiles according to the A/T/N classification scheme among participants with available CSF. A+ refers to abnormal Aβ CSF Aβ42 < 450 pg/ml and/or CSF Aβ42/40 ratio < 1, T+ to abnormal p-tau (CSF p-tau > 60 pg/ml), and N+ to abnormal t-tau (CSF t-tau > 350 pg/ml) and/or MTA (visual rating, score ≥ 1)

Baseline characteristics of the participants with centrally assessed biomarkers (MTA on MRI and/or CSF) are shown in Table 2. Out of 287 participants with at least one centrally analyzed baseline biomarker, 180 (62.7%) had only MTA assessment available; CSF was analyzed for 107 participants (37.3%). Participants with CSF available were younger and had lower CDR-SB. In total, 62.3% of the participants with available apolipoprotein E (*APOE*) data were ε4 carriers. MTA score was at least 1 in 86.4% (241 out of 279) of the participants. CSF Aβ, t-tau, and p-tau levels were abnormal in 87.9% (94 out of 107), 86.0% (92 out of 107), and 69.2% (74 out of 107) of the participants, respectively. Classification according to the A/T/N biomarker scheme is shown in Fig. 1. The majority, 63.6%, had an A+T+N+ profile.

**Table 2:** Baseline characteristics of the study population

Characteristics	***N*** data available	CSF and/or MRI available (***N*** = 287)	Only MRI available (***N*** = 180)	CSF available (***N*** = 107)	***p***-value
**Demographics**					
Age, years	287	70.9 (6.6)	71.6 (6.2)	69.8 (7.0)	0.02
Female	287	147 (51.2%)	100 (55.6%)	47 (43.9%)	0.06
Education, years	287	10.5 (3.7)	10.6 (3.8)	10.4 (3.7)	0.62
**Cognition**					
MMSE score	286	26.6 (2.0)	26.7 (2.0)	26.6 (1.9)	0.94
NTB composite	284	0.012 (0.682)	− 0.010 (0.711)	0.050 (0.630)	0.48
NTB memory	283	0.014 (0.848)	0.021 (0.885)	0.003 (0.786)	0.87
NTB executive functioning	281	0.007 (0.681)	− 0.046 (0.656)	0.095 (0.718)	0.09
NTB total	283	0.004 (0.549)	− 0.003 (0.561)	0.017 (0.532)	0.77
CDR-SB	259	1.76 (1.12)	1.91 (1.17)	1.53 (1.02)	0.01
**Biomarkers and** ***APOE***					
*APOE* ε4 carrier	260	162/260 (62.3%)	97/156 (62.2%)	65/104 (62.5%)	0.96
MTA score	279	2 [1–4]	3 [1–4]	2 [1–3]	0.11
Abnormal MTA	279	241/279 (86.4%)	155/180 (86.1%)	86/99 (86.9%)	0.86
CSF Aβ42, pg/ml	107	412 (242)	NA	412 (242)	NA
CSF Aβ42/40 ratio	107	0.63 (0.27)	NA	0.63 (0.27)	NA
Abnormal Aβ	107	94/107 (87.9%)	NA	94/107 (87.9%)	NA
CSF t-tau, pg/ml	107	616 (276)	NA	616 (276)	NA
Abnormal t-tau	107	92/107 (86.0%)	NA	92/107 (86.0%)	NA
CSF p-tau, pg/ml	107	78 (29)	NA	78 (29)	NA
Abnormal p-tau	107	74/107 (69.2%)	NA	74/107 (69.2%)	NA

Data are mean (SD), median [IQR], or *N* (%). *p*-values are shown for comparisons between participants with only MRI available and those with centrally analyzed CSF. Abnormal MTA defined as a score of ≥ 1; abnormal Aβ as CSF Aβ42 < 450 pg/ml and/or CSF Aβ42/40 ratio < 1; abnormal t-tau as CSF t-tau > 350 pg/ml; abnormal p-tau as CSF p-tau > 60 pg/ml

*Abbreviations: Aβ42*, β-amyloid 1–42; *Aβ40*, β-amyloid 1–40; *Aβ42/40 ratio*, Aβ42/40 × 10; *APOE*, apolipoprotein E; *CDR-SB*, Clinical Dementia Rating-Sum of Boxes; *CSF*, cerebrospinal fluid; *MMSE*, Mini-Mental State Examination; *MRI*, magnetic resonance imaging; *MTA*, medial temporal lobe atrophy, visual rating; *NTB*, Neuropsychological Test Battery; *p-tau*, tau phosphorylated at threonine 181; *t-tau*, total tau

**Fig. 2:**
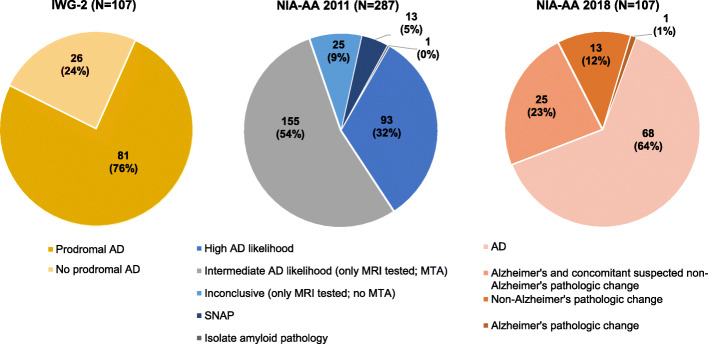
Classification of study participants according to the IWG-2, NIA-AA 2011, and NIA-AA 2018 research diagnostic criteria

Figure 2 illustrates the classification according to the different research diagnostic criteria. In total, 75.7% (81 out of 107) of the participants had IWG-2 prodromal AD. Approximately half of the participants were classified in the NIA-AA 2011 intermediate AD likelihood group (54.0%, 155 out of 287) and 8.7% (25 out of 287) in the inconclusive/uninformative group. A third had a high AD likelihood (32.4%, 93 out of 287) and 4.5% (13 out of 287) were categorized in the SNAP group. One had an isolate amyloid pathology; no one had a low AD likelihood. With respect to the NIA-AA 2018 criteria, 63.6% of the participants (68 out of 107) were classified in the AD group, 23.4% (25 out of 107) in the Alzheimer’s and concomitant suspected non-Alzheimer’s pathologic change group, and 12.1% (13 out of 107) in the non-Alzheimer’s pathologic change group. One had an Alzheimer’s pathologic change. However, even a slight adjustment of the p-tau cut-off (> 55 pg/ml instead of > 60 pg/ml) changed the classification: in this case, more participants were assigned to the AD group (72.0%, 77 out of 107) and fewer participants to the Alzheimer’s and concomitant suspected non-Alzheimer’s pathologic change group (15.0%, 16 out of 107). The proportion of *APOE* ε4 carriers was high in groups with stronger evidence for AD pathology, and the percentage seemed to increase when applying the more recent criteria (NIA-AA 2011 intermediate AD likelihood 64.5%, high AD likelihood 72.2%, IWG-2 prodromal AD 76.9%, NIA-AA 2018 AD 78.5%; data not shown).

### Disease progression

Table 3 shows per each set of criteria the estimates for 2-year change in cognitive and cognitive-functional performance, progression rates to dementia, and hazard ratios (HR) for dementia risk.

**Table 3:** Two-year change from baseline in NTB and CDR-SB scores and progression to dementia according to the IWG-2, NIA-AA 2011, and NIA-AA 2018 criteria

	N with data	Change in NTB composite		Change in NTB memory		Change in NTB executive functioning		Change in NTB total		Change in CDR-SB		Progression to dementia	
		*Estimate*	*p*	*Estimate*	*p*	*Estimate*	*p*	*Estimate*	*p*	*Estimate*	*p*	*N (%)*	*HR (95% CI)*
**IWG-2**													
No prodromal AD	24	−0.018	Ref	−0.004	Ref	−0.085	Ref	0.048	Ref	0.42	Ref	7/26 (26.9)	Ref
Prodromal AD	76	−0.254	0.11	−0.266	0.11	−0.097	0.93	−0.107	0.13	1.18	0.03	34/81 (42.0)	4.6 (1.6–13.7)
**NIA-AA 2011**													
SNAP	13	0.081	Ref	0.128	Ref	−0.044	Ref	0.057	Ref	0.56	Ref	1/13 (7.7)	Ref
Inconclusive/uninformative	22	0.129	0.79	0.087	0.85	0.014	0.75	0.109	0.71	0.21	0.51	4/25 (16.0)	2.0 (0.2–18.7)
Intermediate AD likelihood	135	−0.192	0.07	−0.209	0.06	−0.144	0.50	−0.169	0.05	1.29	0.08	64/155 (41.3)	6.8 (0.9–50.0)
High AD likelihood	86	−0.195	0.08	− 0.186	0.08	−0.083	0.80	−0.084	0.23	1.08	0.23	40/93 (43.0)	7.4 (1.0–54.7)
**NIA-AA 2018**													
Non-Alzheimer’s pathologic change	13	0.057	Ref	0.049	Ref	−0.088	Ref	0.038	Ref	0.48	Ref	1/13 (7.7)	Ref
Alzheimer’s and concomitant suspected non-Alzheimer’s pathologic change	22	−0.154	0.31	−0.125	0.45	−0.112	0.91	0.029	0.96	0.43	0.91	10/25 (40.0)	3.5 (0.4–29.4)
AD	64	−0.274	0.08	−0.299	0.09	−0.087	0.99	−0.134	0.20	1.43	0.03	30/68 (44.1)	9.4 (1.2–72.7)

Estimates for change in NTB and CDR-SB scores are least-squares means for change from baseline over 2 years within each group. Negative values indicate decline over time, except for CDR-SB where positive values indicate decline. *p*-values are shown for the difference in the least-squares means over 2 years between each group and the respective reference group. Numbers of participants are as per modified intention-to-treat (mITT) analysis. CDR-SB data were missing for nine participants (IWG-2, NIA-AA 2018) and 32 participants (NIA-AA 2011). Isolate amyloid pathology/Alzheimer’s pathologic change was excluded from the analyses (*N* = 1)

*Abbreviations: AD*, Alzheimer’s disease; *CDR-SB*, Clinical Dementia Rating-Sum of Boxes; *IWG*, International Working Group; *NIA-AA*, National Institute on Aging–Alzheimer’s Association; *NTB*, Neuropsychological Test Battery; *SNAP*, suspected non-AD pathology

### IWG-2 criteria

The changes in NTB scores were modest overall in both IWG-2 groups, and no differences were observed between the groups (Table 3). For CDR-SB, there was more worsening in the prodromal AD group (estimate for change 1.18 points vs. 0.42 points; *p* = 0.03). This group also had a higher risk of progression to dementia (42.0% vs. 26.9%; HR 4.6, 95% CI 1.6–13.7).

### NIA-AA 2011 criteria

The changes in NTB scores were fairly small in all NIA-AA 2011 groups. No statistically significant differences were observed between the reference group SNAP and the other groups, but there was a trend indicating somewhat higher rate of decline in the intermediate and high AD likelihood groups (Table 3). The pattern of decline in these two groups was however similar. Individuals with a high AD likelihood were more likely to progress to dementia than those with SNAP (43.0% vs. 7.7%; HR 7.4, 95% CI 1.0–54.7). The risk was similar in the high and intermediate AD likelihood groups.

### NIA-AA 2018 criteria

The changes in NTB were small across all NIA-AA 2018 groups. The AD group showed consistently the highest rate of decline, but the changes did not differ from those of the reference group non-AD pathologic change (Table 3). CDR-SB scores worsened more in the AD group than in the reference group (estimate for change 1.43 points vs. 0.48 points; *p* = 0.03) and in the Alzheimer’s and concomitant suspected non-Alzheimer’s pathologic change group (1.43 points vs. 0.43 points; *p* = 0.01). The AD group was also more likely to progress to dementia than the reference group (44.1% vs. 7.7%, HR 9.4, 95% CI 1.2–72.7) and the Alzheimer’s and concomitant suspected non-Alzheimer’s pathologic change group (44.1% vs. 40.0%, HR 2.7, 95% CI 1.0–7.0).

## Discussion

In this study, we examined the baseline biomarker profiles of individuals with IWG-1 prodromal AD enrolled in the LipiDiDiet RCT. We found that in the subset of study participants with available centrally analyzed biomarkers (including CSF), most individuals displayed a clear AD biomarker profile and could be classified as having IWG-2 prodromal AD, high AD likelihood (NIA-AA 2011), and AD (NIA-AA 2018). Approximately 90% of those with centrally analyzed CSF had abnormal Aβ; most participants also had an A+T+N+ profile. In line with studies reporting that the IWG-1 criteria are sensitive and have a decent prognostic accuracy [8, 25], our findings suggest that the IWG-1 criteria might reliably capture an early symptomatic AD population—even if they require less comprehensive biomarker evidence than the more recently proposed criteria. Given that the LipiDiDiet participants—who were primarily memory clinic patients—had amnestic cognitive impairment and often already signs of brain atrophy on MRI, the high prevalence of Aβ positivity and A+T+N+ profile was expected. This is because Aβ accumulation is thought to precede neuronal injury, which in turn correlates closely with clinical symptoms [26]. Among the participants whose CSF was analyzed but not all biomarkers were abnormal, a considerable proportion had CSF p-tau within the normal range and an A+T−N+ profile, indicating either subthreshold tauopathy or amyloidosis in combination with non-AD pathologies [27–29]. In previous studies, the A+T−N+ profile has been uncommon in mild cognitive impairment (MCI), both in memory clinic patients [30, 31] and in more selected cohorts [32, 33]. We observed that even a small adjustment of the p-tau cut-off changed the classification, suggesting that several LipiDiDiet participants with an A+T−N+ profile had subthreshold levels of tau. In a recently published study where data-driven methods were applied to determine unbiased cut-off points for tau, researchers identified three different cut-offs instead of a single clear cut-off, resulting in four subgroups of individuals with somewhat different cognitive trajectories [34]. Collectively, these findings and our results underline the issue with using sharp dichotomous biomarker cut-offs to classify individuals.

To further investigate the applicability of the research diagnostic criteria, we examined LipiDiDiet participants’ disease progression over 2 years. Cognitive and cognitive-functional decline appeared to be more pronounced in the prodromal AD (IWG-2), high and intermediate AD likelihood (NIA-AA 2011), and AD (NIA-AA 2018) groups, but despite some trends, only few statistically significant between-group differences were observed within each set of criteria. The study was likely underpowered to detect these differences. Another explanation for these findings is that the changes, especially in NTB, were modest overall and lower than expected during the first 2 years of the LipiDiDiet trial [17] but closer to the expectation after 3 years [35]. Here, we observed that the changes were fairly small even among those with both abnormal Aβ and neuronal injury markers. With respect to the CDR-SB scores, the increase was more pronounced in the AD group (A+T+N±) than in the Alzheimer’s and concomitant suspected non-Alzheimer’s pathologic change group (A+T−N+), potentially supporting the distinction between tau (e.g., p-tau) and other neuronal injury markers. However, we do not know if the between-group differences within each set of criteria would differ at older vs. younger ages, since the LipiDiDiet participants without CSF were older, and older individuals are also more likely to have mixed pathologies [36].

The participants in the LipiDiDiet trial developed dementia at rates proportional to the certainty of underlying AD, and the risk of progression was higher among those with IWG-2 prodromal AD, NIA-AA 2011 high AD likelihood, and NIA-AA 2018 AD. Progression rates were very similar in these groups, highlighting the overlap between the criteria and these categories. Overall, our findings are consistent with previous studies showing that MCI individuals with abnormal Aβ and neuronal injury (IWG-2 prodromal AD, NIA-AA 2011 high AD likelihood) might have an increased risk of disease progression compared to those with normal/conflicting biomarkers or abnormal Aβ alone [8, 16]. The few available longitudinal studies investigating the NIA-AA 2018 criteria have also reported an increased risk of decline for the AD profiles, both in MCI [30, 33] and among cognitively healthy individuals [29, 37, 38].

In LipiDiDiet, participant eligibility was often evaluated based on MTA rather than CSF assessment, which is why a large group of individuals were classified in the NIA-AA 2011 intermediate AD likelihood group. Notably, we observed that the rate of disease progression was consistently similar in this group and in the high AD likelihood group. This is an encouraging result, given that the assessment of only one biomarker (usually MTA on widely available MRI) is a common scenario. Methods for measuring Aβ are currently invasive (lumbar puncture) and costly (PET), limiting their application in routine clinical practice and in RCTs conducted in diverse settings. While the IWG-2 and NIA-AA criteria might have a higher specificity [8, 25], the IWG-1 criteria could be preferred in some situations, yet further investigation is needed to identify the optimal set of criteria for participant recruitment to prevention RCT. Ongoing validation studies will also show if plasma biomarkers for p-tau and Aβ can help streamline recruitment for future prevention RCTs [39–41].

One advantage of the IWG-1 criteria over the other research criteria is related to the efficiency of recruitment. The use of more restrictive criteria decreases the number of eligible individuals, as shown also in our study. The choice of criteria may also have implications for the representativeness of the study population. We found that the participants who underwent lumbar puncture for CSF analysis were somewhat younger and had a better cognitive-functional performance than those who did not. Reasons for this are unclear, but older participants might have had more often contraindications for the procedure. In one previous memory clinic study, contraindications were indeed often observed among patients who did not undergo routine lumbar puncture [42]. This study did not find any age difference, but the latter group did have a lower MMSE [42]. In LipiDiDiet, a possible explanation for the observed differences is that the study populations could have been slightly different at different sites, depending on local circumstances. Sites where MRI was the preferred method might have had older patients with poorer cognitive-functional performance. Little is known about the impact of selection criteria on participant characteristics in prodromal AD RCTs, but strict biomarker criteria could limit the study population representativeness through exclusion of many older individuals who often have mixed pathologies [36]. Whenever possible considering the nature of the intervention, including such individuals in RCTs is encouraged as they form a large group of potential intervention end users.

### Limitations

The study population was largely Caucasian and a homogenous group of RCT participants recruited primarily from memory clinics. Our findings may thus not be fully generalizable to other populations or settings, and further investigation will be needed in ethnically and geographically more diverse cohorts, ideally with a longer timeframe to assess disease progression. In the LipiDiDiet RCT, the IWG-1 criteria for prodromal AD were used to recruit participants, and the eligibility criteria included evidence for underlying AD pathology based on either imaging or CSF markers (i.e., participants were not required to have both assessments). Therefore, CSF was not available for the whole study population, and the classification according to certain criteria (IWG-2, NIA-AA 2018) was possible only in a subset of participants. Furthermore, as the sample was overall small, and only few participants were classified in the groups reflecting lower certainty of AD (e.g., low AD likelihood, SNAP), we lacked statistical power to detect between-group differences in the longitudinal analyses of disease progression. The smaller than expected cognitive changes over a rather short follow-up period of 2 years could have also affected our results. LipiDiDiet extension studies will shed light on the longer-term cognitive and cognitive-functional trajectories and prognosis in prodromal AD, which could improve the operationalization of the research diagnostic criteria. Another limitation of our study is that the Aβ and tau assessment was based only on CSF. CSF and PET reflect different aspects of pathology [43] and incorporating PET could have affected the classification. Nevertheless, Aβ status was determined based on both Aβ42 and Aβ42/40 ratio, which is an advantage as the latter parameter is potentially a more accurate measure of Aβ pathology [44]. Another major strength of our study was the possibility to investigate the research diagnostic criteria in a real-life RCT setting. This is because all biomarkers were centrally analyzed, and the sampling and assessment protocols were standardized. In multicenter studies, variability introduced by different laboratory procedures and cut-offs is a common challenge. Cognitive testing was also standardized in our study.

## Conclusions

Assessing the applicability of different participant selection criteria is crucial to inform the design and recruitment of future RCTs. Criteria should be restrictive enough to ensure inclusion of the right population with potential to respond to the interventions, yet feasibility and differences in clinical practices are important considerations. Our findings indicate that the IWG-1 criteria might reliably identify individuals with AD pathology, supporting the use of these criteria in certain prevention RCTs targeting pre-dementia stages. Firstly, while complex assessments like CSF and PET are currently necessary to verify biomarker status in Aβ- or tau-targeting RCTs, more pragmatic and easily applicable criteria could be preferred due to feasibility in trials investigating, e.g., non-pharmacological lifestyle-based interventions. These interventions do not necessarily target any specific pathology but exert their effects through multiple mechanisms of action. Secondly, it is important that prevention strategies are tested also in settings where possibilities for comprehensive biomarker testing are limited, and in these situations, pragmatic criteria like the IWG-1 could potentially be considered. At the moment, several multidomain prevention RCTs are being planned and conducted worldwide, many of them within the World Wide FINGERS (WW-FINGERS) network [45].

## Data Availability

LipiDiDiet is an ongoing study. The LipiDiDiet consortium is open to all requests from external researchers for data collected in the trial. The study protocol is publicly available online at http://lipididiet.eu/fileadmin/lipididiet/publications/LipiDiDietStudyProtocol.pdf. Requesters will be asked to submit a study protocol, including the research question, planned analysis, and data required. The LipiDiDiet Trial Steering Committee will evaluate this plan (i.e., relevance of the research question, suitability of the data, quality of the proposed analysis, planned or ongoing LipiDiDiet analysis, and other matters) on a case-by-case basis and provide the data or reject the request. Shared data will encompass the data dictionary and de-identified participant data only. Any analysis will be conducted in collaboration with and on behalf of the LipiDiDiet consortium. Access is subject to the LipiDiDiet legal framework. An access agreement will be prepared and signed by both parties.

## Abbreviations

Aβ: β-amyloid
Aβ42: β-amyloid 1–42
Aβ42/40: Ratio of β-amyloid 1–42 and β-amyloid 1–40
AD: Alzheimer’s disease
APOE: Apolipoprotein E
A/T/N: Amyloid/tau/neuronal injury classification
CDR-SB: Clinical Dementia Rating-Sum of Boxes
CERAD: Consortium to Establish a Registry for Alzheimer’s Disease
CSF: Cerebrospinal fluid
DSM-IV: Diagnostic and Statistical Manual of Mental Disorders 4th edition
FDG-PET: Fluoro-deoxy-glucose-positron emission tomography
IWG: International Working Group
MCI: Mild cognitive impairment
mITT: Modified intention-to-treat
MMSE: Mini-Mental State Examination
MRI: Magnetic resonance imaging
MTA: Medial temporal lobe atrophy
NIA-AA: National Institute on Aging–Alzheimer’s Association
NINCDS-ADRDA: National Institute of Neurological and Communicative Disorders and Stroke–Alzheimer’s Disease and Related Disorders Association
NTB: Neuropsychological Test Battery
PET: Positron emission tomography
p-tau: Phosphorylated tau protein (at threonine 181)
RCT: Randomized controlled trial
SNAP: Suspected non-AD pathology
t-tau: Total tau protein
WMS-r: Wechsler Memory Scale-revised
WW-FINGERS: World Wide FINGERS
